# The Role of Skin Immune System in Acne

**DOI:** 10.3390/jcm11061579

**Published:** 2022-03-13

**Authors:** Ewelina Firlej, Wioleta Kowalska, Karolina Szymaszek, Jacek Roliński, Joanna Bartosińska

**Affiliations:** 1 Department of Cosmetology and Aesthetic Medicine, Medical University of Lublin, 20-093 Lublin, Poland; joannabartosinska@umlub.pl (J.B.); 57471@umlub.pl (K.S.); 2 Department of Clinical Immunology, Medical University of Lublin, 20-093 Lublin, Poland; jacek.rolinski@umlub.pl

**Keywords:** acne vulgaris, AMP, MMP, Th1 and Th17 lymphocytes, treatment

## Abstract

Acne vulgaris is a skin disease that often occurs in adolescence and in young adulthood. The main pathogenic factors are hyperkeratinization, obstruction of sebaceous glands, stimulation of sebaceous gland secretion by androgens, and bacterial colonization of sebaceous units by *Cutibacterium acnes*, which promotes inflammation. Little is known about the role of skin immune cells in the development of acne lesions. The aim of the study was to try to understand the role of skin immune cells in the course of acne. Recent studies have shown that there are at least four major pathways by which *Cutibacterium acnes* interacts with the innate immune system to induce inflammation: through TLRs, activating inflammasomes, inducing the production of matrix metalloproteinases (MMPs), and stimulating antimicrobial peptide (AMP) activity. Cells of adaptive immune response, mainly Th1 and Th17 lymphocytes, also play an important role in the pathogenesis of acne. It is worth emphasizing that understanding the role of the skin’s immune cells in the pathogenesis of acne may, in the future, contribute to the application of modern therapeutic strategies that would avoid addiction to antibiotics, which would alleviate the spectrum of resistance that is now evident and a current threat.

## 1. Introduction

Acne vulgaris is a skin condition commonly affecting adolescents and young adults. The main pathogenic factors of acne include hyperkeratinisation, the secretion from the sebaceous glands stimulated by androgens, dysbiosis of the skin microbiome, and *Cutibacterium acnes* (*C. acnes*); and the overcolonisation of pilosebaceous units, which might provide favourable conditions for perifolliculitis. The clinical presentation of acne ranges from its mild form (mild comedonal acne) to severe inflammatory nodulocystic acne affecting the skin of the face, chest, and back. Based on current findings, it can be stated that the increased activity of sebaceous glands induced by androgens triggers the proliferation of *C. acnes*, anaerobic bacterium present in the sebum accumulating in follicular ducts. The induction of inflammatory reactions by *C. acnes* is the main aetiologic factor contributing to the pathogenesis of acne vulgaris. In particular, the IL-1 family of cytokines plays a key role both in triggering acne lesions and in the inflammatory response in acne. Nonetheless, there is still a lot to be discovered on the importance of the skin immune cells for the development of acne lesions, both in the early stage and in further stages of acne [1,2].

Saurat J.-H. et al. [3] provides models for further understanding the biological events of comedogenesis. The latest knowledge is gained from both lineage tracking experiments in mice and sebaceous responses to xenobiotics in humans. Emphasis is placed on sebaceous stem cells LRIG1+ in the isthmus of the sebaceous duct. LRIG cells can differentiate toward epithelial or sebaceous type and should, therefore, be prime targets for comedogenic factors. This population can, therefore, be a source of blackhead lesions. This may also explain the sentence that “the more comedones, the fewer mature sebocytes” in acne histology [3,4].

Manfredini M. et al. [5] assessed the evolution of acne lesions from clinically unchanged skin in mild to moderate acne patients using in vivo reflection confocal microscopy (RCM) and dynamic optical coherence tomography (D-OCT). After analyzing seventy complete sets of clinical pictures comrpising RCM and D-OCT, it was shown that the appearance of an acne lesion is preceded by the growth of large follicles in the area corresponding to funnel keratinization. There is an increase in inflammatory parameters, such as the growth of small bright cells in RCM and the vascular network in D-OCT, that return to normal after acute inflammation has subsided [5].

The objective of the paper is to explore the role of the cells of the skin immune system in the course of acne. The paper includes a review and analysis of the literature on the subject published in the period between 2005 and 2021. Without doubt, a better understanding of the pathogenesis of acne might further translate into effective treatments of patients suffering from this skin disease. Acne requires a multidirectional approach and cooperation between physicians specializing in various medical fields, as well as psychologists and cosmetologists. Therapeutic success may be achieved thanks to the understanding and processes accounting for immunology processes taking place in skin and hair follicles.

## 2. The Aetiology and Pathogenesis of Acne

Acne affects 80 percent of adolescents, yet the condition often persists until early adulthood and may cause scarring and skin discolouration [6,7,8]. Acne lesions develop in hair follicles on the skin in the areas of cheeks, forehead, chin, mandible, and chest. The combination of increased secretion of the sebum and abnormal excessive proliferation of keratinocytes results in the development of microscopic lesions called microcomedones. The accumulation of the sebum, the enlargement of a sebaceous gland, and the accumulation of the keratin material in the microcomedone area result in the development of comedones. According to the conventional views of acne pathogenesis, *C. acnes*, which is present on healthy skin, colonize follicular ducts and trigger an innate immune response, resulting in the progression from so-called non-inflammatory comedones to inflammatory papules, pustules, or nodules. However, in the last decade, several pieces of evidence were provided, suggesting that inflammation can occur across the entire life cycle of acne lesions even before comedones develop [7].

**Figure 1:**
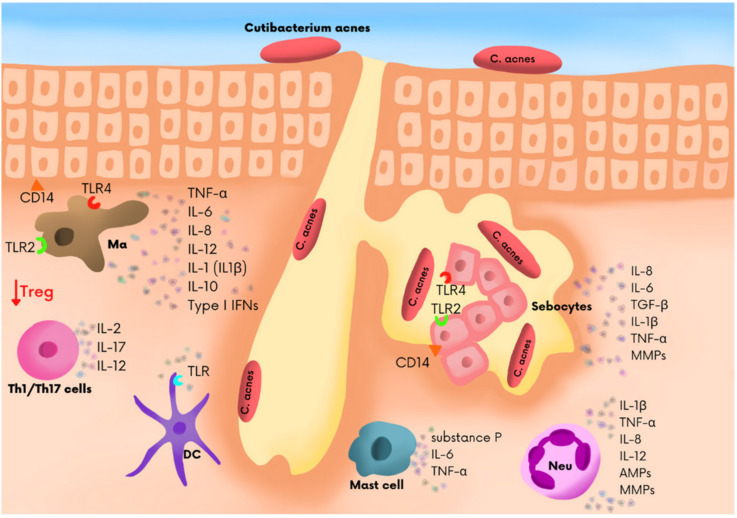
The immune cells in microenvironment of sebaceous gland in acne vulgaris patients. The main skin immune cells, i.e., Langerhans cells, dendritic cells of the dermis, macrophages, mast cells, B and T cells, and keratinocytes [13]. Neutrophils are not cells residing in the skin, but they accumulate in the skin in the course of inflammation. In general, skin cells communicate by secreting large quantities of biologically active cytokines and chemokins, which regulate their function and migration in specific skin layers.

Recent studies conducted at molecular and cell level provided a hint of an explanation of how the factors causing acne interact. Inflammation was found in all acne lesions, and for that reason, an inflammation in the area of the pilosebaceous unit can be regarded as a defining characteristic of acne [9]. The acne exposome, which is the total sum of exposure to all environmental factors, also affects the presence, aggravation, and duration of acne. Exposome-related factors include nutrition, psychological and work-related factors, lifestyle, cosmetics, medications, climate-related factors, and pollution. The factors impact on the skin through the interaction with the skin protective barrier, sebaceous glands, innate immunity, and skin microbiota [10]. It was formerly believed that acne skin lesions were developed upon the abnormal sloughing of keratinocytes that line the sebaceous gland, which resulted in excessive keratosis of the pilosebaceous duct and the formation of microcomedones. However, recent years have experienced a shift in the understanding of acne pathogenesis, and it is currently believed that it is a primary inflammatory dermatitis. Studies showed the presence of a subclinical infection in normal skin of patients with acne, before microcomedones developed [11]. Acne lesions are initiated by an obstruction of the opening of the pilosebaceous duct, which might result from the excessive keratosis of the epithelium. The cells accumulate in the sebaceous gland and move to the pilosebaceous duct. Sebaceous glands expand and sometimes burst, causing the escape of contents to the surrounding tissue. The studies showed the presence of inflammation markers in the contents of microcomedones. It was also shown that IL-1 was present in comedonal contents. It is worth noting that the so-called “inflammatory components”, i.e., CD4+ T cells and macrophages, are also present in the skin and are unaffected by acne lesions [9]. In the course of inflammation, myeloid cells respond immediately and produce pro-inflammatory mediators that affect the activation of cells located in the closest proximity and facilitate the migration of cells to the area of the ongoing inflammatory process [12]. Myeloid cells of the skin also serve as a connection between innate and adaptive immune responses (Figure 1).

Depending on the morphological characteristics and the intensity of lesions, the following clinical forms of acne were identified: comedonal acne, with the prevalence of closed and/or open comedones; papulopustular acne, characterized by the presence of inflammatory lesions (papules and/or pustules); highly inflammatory acne (including nodular acne and conglobate acne) with the presence of nodules or cysts; acne keloidalis, characterized by scarring; acne fulminans; and acne excoriée [13,14,15]. The aforementioned disorders were diagnosed on the basis of medical examination and interview both in the clinical and cosmetology practice. As regards clinical practice, acne belongs to most frequent dermatological diseases, and its pathogenesis requires further research [16,17].

Inverse acne (acne inversa, hidradenitis suppurativa; HS) is a severe, chronic inflammatory disease. Normally, the lesions are located in the area of skin folds and are painful nodules with a tendency to fistula formation and scarring. There are discussions to this day as to whether the disease belongs to the acne group. In the course of the disease, hyperkeratosis and destruction of the hair follicles occur, and the involvement of apocrine sweat glands is a secondary phenomenon. The estimated prevalence of HS is 1%, with a predominance of women [18,19].

PAPA syndrome, a hereditary autoinflammatory syndrome caused by mutations in the proline-serine-threonine phosphatase 1 (PSTPIP1) gene, is clinically characterized by purulent arthritis, acne, and pyoderma gangrenosum. PASH and PAPASH syndromes are similar to PAPA syndrome, but the former does not present accompanying arthritis and has a different genetic background, while the latter is characterized by purulent arthritis, acne, pyoderma gangrenosum, and suppurative hidradenitis. PAPA, PASH, and PAPASH are associated with the over-expression of IL-1 and tumour necrosis factor (TNF)-α and may respond to biological drugs that specifically target these cytokines [20,21,22]. 

## 3. Immune Cells Participating in the Pathogenesis of Acne

The pathogenesis of acne vulgaris is complex and involves mutual interactions between hormonal, microbiological, and immunological factors. Nevertheless, the mechanisms behind the causes of acne vulgaris still remain unclear.

The knowledge adapted in the last decade indicates that acne vulgaris is a disease characterized by excessive inflammation in the area of pilosebaceous units and the surrounding skin. It is partially generated by the interaction of *C. acnes* with the cell components of skin, namely keratinocytes, sebocytes, and tissue macrophages. *C. acnes* is a Gram-positive anaerobic bacterium secreting inflammatory substances that can be crucial in acne pathogenesis [23]. The bacteria produce lipase, hyaluronidase, and proteases and activate immune cells, thus causing inflammation [24]. The substances are identified by PRRs. TLRs constitute one of the main categories of PRRs [23]. Various types of TLR-expressing cells are present in the epidermis, including keratinocytes and Langerhans cells. Cells present in the dermis and migrating to this skin layer also demonstrate TLR expression. These include macrophages, dendritic cells, lymphocytes, and mast cells. In a good state of health, immune cells account for 7% of skin cells, of which ca. 3.78% are Langerhans cells, 0.45% are NK cells, 0.24% are macrophages, 0.79% are dendritic cells, 0.41% are T cells, and 1.33% are other immune cells, i.e., innate lymphoid cells (ILC), neutrophils, and macrophages. It is worth noting that 80% of all T cells residing in the skin are memory T cells. CD4+ and CD8+ T_RM_ cells account for 83% and 17% of T cells, respectively, while the share of regulatory T cells (Treg) amounts to approximately 5–10% of all T cells residing in the skin. Yang et al. [25], in their studies, found a high percentage of wandering neutrophils, monocytes, and activated mast cells but a lower infiltration of Treg cells in areas affected by acne lesions. Interestingly, the scholars observed a significantly higher infiltration of Treg cells and resting dendritic cells in non-lesional skin areas of acne patients in relation to the skin of healthy individuals. This suggests immunosuppression in unaffected areas of the skin of acne patients, which might explain, i.e., the absence of papules there. In their studies, Ozlu et al. [26] identified TLR2+ macrophages in acne lesions and around pilosebaceous units and proved that the number of the cells increased as the condition progressed. In turn, Jugeaou et al. [27] described an upregulated expression of TLR2 and TLR4 in keratinocytes in persons with inflammatory acne. It is suspected that, in the course of inflammatory acne, *C. acnes* activates cells by interactions with TLR2 and TLR4. This interaction results in the production of pro-inflammatory cytokines including TNF and IL-1, and IL-8 and Il-12. IL-8 contributes to the recruitment of neutrophils and further damage to the epithelium of sebaceous glands, whereas IL-12 promotes Th1-type response [28]. It was also showed that, as acne was aggravated, the cells demonstrated an increased expression of TLR2. TLR2, also present on the surface of perifollicular macrophages, shows affinity to *C. acnes*, which triggers the secretion of pro-inflammatory cytokines, such as IL-8 and IL-12. These interleukins also create favourable conditions for the chemotaxis of neutrophils and the secretion of lysosome enzymes that take part in the mechanism of intracellular bacteria killing. This probably explains why a higher efficacy to TLR2-targeted drugs, such as topical retinoids, was observed in patients with a more severe form of acne [29]. TLR2 seems to be an effective target for therapeutic intervention based on blocking the inflammatory response in the course of the invasion of *C. acnes*. Therefore, research focused on TLR2 provides valuable information on new therapeutic targets of acne vulgaris. It was also proven that *C. acnes* might also induce the differentiation of monocytes into two separate cell subsets of innate immune response: CD209+ macrophages that more effectively phagocytose and kill *C. acnes* and CD1b+ dendritic cells that activate T cells and release pro-inflammatory cytokines [28]. It is worth stressing that the synthesis of chemokines and cytokines by monocytes is induced by the activation of TLR2 in which *C. acnes* is mediated [26]. Jugeau et al. [27], in their studies aimed at defining the role of *C. acnes* in inflammatory acne, showed an increased expression of TLR2 and TLR4 in the epidermis of acne lesions in vivo. In turn, Selway et al. [30] evaluated the role of TLR2 in the pathogenesis of acne and its significance in comedogenosis. The scholars found the expression of TLR2 in basal and infundibular keratinocytes and sebaceous glands and discovered that its activation provoked the release of IL-1α from primary human keratinocytes in vitro [30]. It was also shown that the increased activation of TLR2 stimulated excessive cornification and comedogenosis in human sebaceous glands ex vivo [26]. Prior studies had shown that some medications applied in the treatment of acne might modify the expression of TLR. In a study performed by Tenaud et al. [31], it was proven that, in acne patients, the expression of TLR2 by keratinocytes was downregulated after 24 h of incubation with adapalene. In turn, Jarrousse et al. [32] found that zinc salts were able to reduce the expression of TLR2, thus demonstrating anti-inflammatory action in the course of acne [26]. Jeoung et al. [33] examined the influence of topical ALA-photodynamic therapy for acne on the level of TLR2 and TLR4 expression. In this study, five out of 10 cases had elevated TLR2 expression before photodynamic therapy. The expression of this receptor was reduced after the application of the therapy. However, no changes in patients with normal immunoreactivity of TLR2 were reported. It was also shown that the expression of TLR4 was increased prior to the therapy in three out of ten patients, and the level of TLR4 expression decreased after therapy was completed [26,33].

In their studies, Bakry et al. [34] showed significant differences in the intensity of TLR2 expressions in pilosebaceous units and inflammatory infiltrations of the skin between acne-affected skin and normal skin. It was found that the expression of TLR2 was more intense in pilosebaceous units and skin infiltrations in areas of inflammatory lesions and severe acne lesions [34]. Moreover, Ozlu et al. [26] showed that the expression of TLR increased in papular and comedonal lesions in relation to nodular lesions in the epidermis area and in papular lesions in comparison with pustular lesions in the inflammation region and in the dermis area. Moreover, TLR4 expression was lower in comedonal lesions than in papular lesions in the dermis [26]. The results suggest that TLRs play a significant role in the development of various clinical presentations of acne. The inflammatory response triggered by *C. acnes*, including the secretion of IL-1 cytokines, constitutes the key pathogenic factor resulting in the manifestation of the disease. *C. acnes* promotes the secretion of IL-1β and IL-18 through the inflammasome pathway with caspase-1 mediation and nucleotide-binding oligomerization domain (NLRP), such as sensor protein. Inflammasomes are a group of intracellular proteins that transform pro-caspase-1 into caspase-1. Caspase-1 activates pro-interleukin-1β to its active form [29]. It is worth noting that both NLRP3 active caspase-1 are expressed by tissue macrophages CD86+ in acne lesions, which also suggests the participation of inflammasome complexes in acne pathogenesis. The exact mechanism related to the activation of NLRP3 inflammasome is still unclear. However, Qin et al. [35] showed in their research that the K+ efflux is necessary to activate NLRP3-dependend caspase-1 in human monocytes and, consequently, to the further release of IL-1β in response to stimulation by *C. acnes*. In light of the potential role of IL-1β in acne pathogenesis, monoclonal anti-IL-1β and/or molecules that could regulate NLRP3, caspase-1, or K+ efflux should be considered in treatments of acne [35].

Additionally, *C. acnes* stimulates the production of matrix metalloproteinases (MMP). These enzymes are related to inflammation and may play a vital role in matrix degradation and in the formation of post-acne scarring [29]. Numerous studies have shown that *C. acnes* triggers the increased activity of several MMPs [29,36]. A few types of MMPs were found in the sebum of acne patients, including MMP-1, MMP-13, and MMP-9. In their studies, Hammam et al. [36] observed that the level of MMP-9 in the blood samples taken from acne patients was significantly higher relative to the control group. Moreover, the scholars observed that the relationship between the level of MMP-9 and the number of face areas affected by inflammatory pustules and nodules, but no interrelation was found between MMP-9 and scarring [36].

Research performed in recent years have demonstrated that antimicrobial peptides (AMP) play a significant role in the pathogenesis of chronic inflammatory skin diseases [26,37]. AMPs are an important component of the innate immune system. AMPs are structures that both ensure basic protection and induce the development of the immune system. They are recognized on the basis of their cutaneous antimicrobial and immunomodulatory properties and demonstrate the ability to inhibit not only bacterial, protozoal, and fungal infections but also viral infections [38]. It is believed that these proteins play their antimicrobial function by binding with the surface of microorganisms and forming pores on their cell membranes. The AMP family includes α- and β-defensins, S100 protein, ribonuclease, and others [26]. Other AMPs, such as β-defensin (hBD)-2 and hBD-3, cathelicidin LL-37 show a low level of expression in healthy skin, but they are induced in the course of a skin inflammation and infection. The release of AMP may be induced by bacteria, bacterial products, TLR, or pro-inflammatory cytokines [39]. In addition to antibacterial action, AMPs are also strongly engaged in the migration of inflammatory cells and the release of cytokines [26]. In recent years, it was proven that the regulation of AMP synthesis was disturbed in acne patients. In in vitro studies, it was observed that *C. acnes* induce the expression of hBD-2 by keratinocytes and sebocytes. Given that, the increased expression of hBD-2 in acne lesions might be triggered by *C. acnes*. There is evidence that TLRs, such as TLR2 and TLR4, for which their expressions are upregulated by *C. acnes*, could mediate in induction. Cathelicidin LL-37 is another AMP demonstrating activity against *C. acnes* in in vitro conditions. With respect to acne pathogenesis, it has been found that LL-37 limits bacterial growth and shows anti-inflammatory properties; for instance, LL-37 is able to reduce the production of TNF by macrophages stimulated by bacterial components [24]. Medications applied in acne therapy can also modulate AMP expression. Using the ex vivo lipopolysaccharide LPS-induced inflammatory skin explant model, Poiraud et al. [40] demonstrated an increased expression of hBD2 following treatment by zinc gluconate, but no changes were found in hBD4 expression [40]. Boroyava et al. [41] examined the effect of isotretinoin therapy in acne on AMP expression levels with the use of RT-PCR. The scholars measured AMP expression levels before, during, and six months after the commencement of isotretinoin therapy. In relation to the control group, an increased expression of, i.e., cathelicidin, HBD-2, lactoferrin, and lysozyme, and lower expression levels of α-defensin-1 were observed in acne patients prior to the therapy. The expression of cathelicidin, HBD-2, and lactoferrin was reduced in the course of therapy. On the other hand, isotretinoin therapy had no effect on the increased expression of lysozyme and ribonuclease-7, which demonstrate a stronger antimicrobial action than pro-inflammatory action [40,41]. Without doubt, the antibacterial action of AMPs in acne will have a beneficial effect, which has been proven in the application of standard antibiotics and other antibacterial agents in the treatment of acne.

In recent years, emphasis was also placed in the importance of adaptive immune response cells in the course of acne. It was proven that immunogenic proteins of *C. acnes* released to the sebaceous gland duct may be processed by Langerhans cells, which in turn may present antigens to CD4+ T cells in local lymph nodes. Histological studies showed that CD4+ T cells were the most numerous cells of the white blood cell system in early-stage inflammatory infiltrations (6–72 h) in acne lesions, which suggests that they might take part in immune response triggered by the colonization of the sebaceous gland by *C. acnes* [42]. Neutrophils appear in these lesions after 24 and 72 h, when such lesions are clinically identified as pustules. CD8+ cells infiltrate the lesions at a later stage [43]. Recent in vitro studies have shown that *C. acnes* also stimulate adaptive immune response, by inducing Th1 and Th17 lymphocytes to secrete IFN-γ and IL-17A and other pro-inflammatory cytokines [29]. In addition, IL-17^+^ cells were found in perifollicular infiltrations in biopsy specimens of inflammatory acne lesions [44]. It is worth noting that, cytokines, i.e., IL-1β and IL-6 i TGF-β, were also found in acne lesions, and they play a significant role in the activation of Th17. Hence, acne was called T helper type 17 (Th17)-mediated disease [42]. Th17 cells not only characteristically induce the recruitment of neutrophils, which contribute to antimicrobial action, but also cause tissue injury [44]. In the same studies, it was shown that vitamin A (all-trans-retinoic acid) and vitamin D (1,25-dihydroxyvitamin D3) supressed this inflammatory stimulation. This discovery might be used to develop another acne therapy option in the future. Kistowska et al. [42] also found that *C. acnes* can promote mixed Th17/Th1 responses by inducing the secretion of IL-17A and IFN-γ from specific CD4+ T cells in vitro. Furthermore, it has been shown that both *C. acnes*-specific subpopulations of Th17 and Th17/Th1 cells can be found in the peripheral blood of patients suffering from acne [42]. Kelhala et al. [45] took an attempt to examine an inflammatory response, particularly the IL-23/Th17/IL-17A axis in acne lesions in vivo [45]. IL-17A and IL-17F are the key cytokines for the recruitment and activation of neutrophils, but they can also target different cell types including keratinocytes, endothelial cells, monocytes, and fibroblasts. These cells have the ability to secrete pro-inflammatory mediators, i.e., IL-6, TNF, IL-1β, PGE2, and MMP. All the aforementioned cytokines activate the production of Th17 from naive T cells in the presence of IL-23. TGF-β is a key cytokine in both Th17 and Treg-cell differentiation, while IL-17 and IFN-γ synergize in pro-inflammatory cytokine production in keratinocytes [45]. It is worth noting that Treg cells have not been studied in the context of acne lesions before. Kelhala et al. [45] were the first ones to demonstrate that the number of Foxp3+ cells significantly increased in acne lesions in the papillary dermis based on the results of immunohistochemistry. Regulatory T cells prevent autoimmunity and suppress immune responses. Retinoic acid can regulate reciprocally Tregs and Th17 via TGF-β-dependent generation of Foxp3. It is believed that it is a mechanism that may be of importance in the treatment of acne by isotretinoin. Moreover, elevated serum levels of IL-10 were found in acne patients, and the expression of this cytokine was increased in acne lesions. Although acne is often a chronic disease, a single acne lesion is seldom secondarily infected and is rapidly demarcated. The increased IL-10 expression and Treg cells may demarcate the inflammation of a single acne lesion efficiently [44]. The data provided indicate that acne vulgaris is a primary infectious disease, and histological, immunological, and clinical evidence suggest that inflammation is present at all stages of acne-lesion development [7]. Immune pathways at the core of initiating and propagating inflammation in acne are complex and may be related to *C. acnes*. Furthermore, inflammation may occur in acne lesions independently of these bacteria. The process is mediated by androgens or by neurogenic activation, followed by the secretion in the skin of pro-inflammatory neuropeptides [45,46].

## 4. Genetic Factors in Pathogenesis Acne Vulgaris

Acne vulgaris is a disease for which its occurrence and severity depend on various factors. These include, e.g., demographic, diet and lifestyle, and hormonal and genetic factors [47]. Current scientific reports on genes related to clinical symptoms and acne severity are scarce. Recent genome-wide studies have shown that genes associated with acne influence sebaceous gland functions or the development of an inflammatory immune response [48,49]. The *TNF* gene variants located on chromosome 6p21.33 are the most frequently studied genes in terms of the severity of acne lesions caused by pro-inflammatory immune responses [47]. The studies present that the crucial variants are variability in positions -308, -238, -863, -857, and -1031 TNF SNP in acne [50,51]. The polymorphism of genes for interleukins (IL) such as *IL1A*, *IL1B*, *IL4*, *IL6*, *IL8*, *IL10*, *IL17A*, *IL17F*, and related antagonists and receptors *IL1RN*, *IL4R*, *IL17RB*, and *IL23R* family genes are also responsible for the develop inflammation during acne vulgaris [47].

The groups of genes related to acne and a serious risk of acne can affect the function and activity of sebaceous glands. The families of the cytochrome P450 (CYP) gene and the 3-β HSD family (HSD3B) are two frequently examined families in analysis with respect to acne vulgaris [47,52,53].

## 5. Targeted Therapeutic Strategies in Acne Vulgaris

Targeted therapy for acne vulgaris is still under research and clinical trials. Recent studies report that agents that may be used to treat acne act by modulating the immune system.

One of these therapeutics include the inhibitors of interleukin-1β signaling, for example, Gevokizumab (XOMA 052) [54,55]. Gevokizumab is an anti-Il-1β humanized monoclonal immunoglobulin IgG2 antibody. Tle therapeutic role of Gevokizumab in acne vulgairs is actually studied in clinical trials (ClinicalTrial.gov Identifier: NCT01498874) [54,55]. IL-1β plays an important role in pathogenesis of acne vulgaris. The trial suggests that the inhibitors of interleukin-1β could be potentially used in acne treatment in the future.

## 6. Conclusions

Acne is a multifactorial immune-mediated chronic inflammatory disease of the pilosebaceous unit. *C. acnes*, which is present in sebaceous glands both in acne patients and in healthy individuals, plays a critical role in triggering host immune responses, which are considered crucial to the pathogenesis of acne vulgaris and are responsible for the clinical signs of acne vulgaris. There are at least four main pathways via which *C. acnes* interacts with the innate immune system to induce an inflammation: via TLRs, the activation of inflammasomes, the induction of the generation of matrix metalloproteinases (MMP), and the stimulation of antimicrobial peptides (AMP). Specific immune response cells, mainly Th1 and Th17 cells, also play a significant role in the pathogenesis of acne.

It is worth stressing that, in the future, the full understanding of the role of skin immune cells in acne pathogenesis might contribute to the development of state-of-the-art therapeutic strategies.

## Data Availability

Not applicable.
